# Point-of-Care Blood Testing in Severe Mental Illness: A Mixed-Methods Evaluation

**DOI:** 10.1192/bjo.2024.198

**Published:** 2024-08-01

**Authors:** Monty Lyman, Jack Fanshawe, Josh Brewin, Thomas Fanshawe, Philip Turner

**Affiliations:** 1 Department of Psychiatry, University of Oxford, Oxford, United Kingdom; 2 Nuffield Department of Primary Care Health Sciences, Oxford, United Kingdom

## Abstract

**Aims:**

There is a significant mortality gap between the general population and people with SMI. This is especially prominent in those with psychotic disorders, underpinned by an increased risk of cardiometabolic disease. Identifying patients at risk early in their psychotic disorder is of key importance to reduce this mortality gap. Despite the recognised importance of regular physical health assessments in this group, completion rates are suboptimal. Point-of-care testing (POCT) to screen for diabetes and hyperlipidaemia, providing a result from a fingerprick sample in under 10 minutes presents a potential solution to enhance delivery of physical health checks and improve health outcomes in a proactive manner.

We introduced POCT across EIP teams in Southeast of England and evaluated the impact on physical health check completion rates and the quality of clinician-patient interactions in EIP teams.

**Methods:**

A stepped wedge study was performed, introducing Abbot Afinion-2 machines across 30 EIP teams in all eight Mental Health Trusts in South East England (2021–2022). Numbers of completed physical health checks, and HBA1C and lipids blood tests completed in six months before and six months after introduction of POCT were collected from individual patients. Data were compared with those from the South West, which acted as a control region. Data were analysed from National Clinical Audit of Psychosis (NCAP) over comparable date range (2021–2022) to corroborate the findings. Clinician questionnaires were administered at three timepoints (after training, two-months, and eight-months), capturing training experiences, device usability and impacts on patient interactions.

**Results:**

In Southeast England, the rate and quality of physical health checks increased after introduction of POCT HbA1c testing OR 2.02 (95% CI 1.17 to 3.49), lipids 2.38 (1.43 to 3.97), and total completed health checks 3.61 (1.94 to 7.94). These increases were not seen in the Southwest region that did not introduce the machines. A post-hoc review of national audit data also showed a greater improvement of health checks in the intervention group compared with the comparator group over an overlapping timescale. Findings from the questionnaires evidenced improved patient engagement, clinician empowerment and the preference of POCT over traditional blood tests in this setting.

**Conclusion:**

POCT is associated with improvements in the rate and quality of physical health checks, and this study emphasizes the potential of POCT in reducing health inequalities and enhancing holistic care for individuals living with severe mental illness.

